# Where Sepsis and Antimicrobial Resistance Countermeasures Converge

**DOI:** 10.3389/fpubh.2017.00006

**Published:** 2017-02-06

**Authors:** Timothy J. J. Inglis, Nadia Urosevic

**Affiliations:** ^1^The Marshall Centre for Infectious Diseases Training and Research, School of Biomedical Sciences, University of Western Australia, Perth, WA, Australia; ^2^Department of Microbiology, PathWest Laboratory Medicine WA, Queen Elizabeth II Medical Centre, Nedlands, WA, Australia

**Keywords:** antimicrobial resistance, sepsis, integrated systems biology, biocomplexity, microbial forensics, infection control

## Abstract

The United Nations General Assembly debate on antimicrobial resistance (AMR) recognizes the global significance of AMR. Much work needs to be done on technology capability and capacity to convert the strategic intent of the debate into operational plans and tangible outcomes. Enhancement of the biomedical science–clinician interface requires better exploitation of systems biology tools for in-laboratory and point of care methods that detect sepsis and characterize AMR. These need to link sepsis and AMR data with responsive, real-time surveillance. We propose an AMR sepsis register, similar in concept to a cancer registry, to aid coordination of AMR countermeasures.

## Introduction

The United Nations high-level meeting on antimicrobial resistance (AMR) was calculated to thrust the issue of AMR into public view (1) and represents the latest milestone in a global awareness-raising campaign by public health authorities. At first glance, this appears to be the antithesis of precision public health, which places an emphasis on targeted multidisciplinary application of emerging biotechnology to the specific health needs of individuals (2). However, this onslaught against a leading global health challenge is built on a foundation of laboratory AMR surveillance and powered by similar multidisciplinary application of emerging high-throughput biotechnologies (3). The big data outputs obtained in such a way are attractive to public health precisely because they are amenable to mathematical modeling of the ecological and evolutionary processes that lead to AMR (4). These dynamic aspects of infection are complex and have led to a widening comprehension gap. Consequently, the growing public recognition of AMR has yet to acquire a more sophisticated understanding of its personal implications (5, 6). Health professionals who share our concern about escalating AMR support the translation of global policy into action at local, national, and international levels (7). A global campaign to contain and control AMR needs translation from strategic policy into day-to-day health-care practice. Strategy; the practice of the art of war by the *strategos* or general, includes the broader considerations of game theory, complexity, business, and management strategy (8). Biocomplexity provides an attractive framework for placing the cell and molecular biology or biomedical end of the AMR scale in a broader context that includes the clinical pathology of tissues and organs, and ultimately population health including all professional, social, and government regulation (9). So, to understand the mechanistic workings of an emerging public health phenomenon such as the rise in AMR infections, it is necessary to descend the scale of biological organization from population health to the molecular and cellular mechanisms of multiple-drug resistance in different bacterial species (10). A robust assessment of the broad consequences of AMR requires the converse; an ascent from a specific AMR phenotype to multinational surveillance review (11, 12). An unavoidable feature of AMR is its capacity for unpredictable double transmission: the ability to not only enhance case-clusters of transmissible disease, but also for transmission between resistant and previously sensitive bacteria contributing to novel disease case-clusters, as seen in the dissemination and proliferation of multiple mechanisms of carbapenem resistance (13). Both specific mechanisms and means of AMR transmission need consideration, since both the AMR mechanism and its transmission will impact on the ecology and epidemiology of AMR infection and have implications for the measures needed to control AMR (14). New analytical systems biology tools provide scope for evidence-based design of AMR surveillance and control (15). The complex picture that emerges can be used to develop an AMR narrative that covers the wide range of AMR molecular signatures, multiple bacterial species, and AMR mechanism combinations across the broad scale of biological organization (3). However, other emerging systems biology methods such as proteomics, metabolomics, and bacterial cytomics have yet to be integrated in a holistic AMR analysis that forms a more compelling argument for a specific causal effect (16). Practical use of this approach to attribution of causality has been explored in the field of microbial forensics and has wider application in linking the different tiers of analysis up to a strategic level (17). The O’Neill Review identified critical vulnerabilities that could be exploited in control of the global AMR problem and made a series of recommendations (18):

1. A massive global public awareness campaign,
2. Improve hygiene and prevent the spread of infection,
3. Reduce unnecessary use of antimicrobials in agriculture and their dissemination into the environment,
4. Improve global surveillance of drug resistance and antimicrobial consumption in humans and animals,
5. Promote new, rapid diagnostics to cut unnecessary use of antibiotics,
6. Promote development and use of vaccines and alternatives,
7. Improve the numbers, pay and recognition of people working in infectious disease,
8. Establish a Global Innovation Fund for early-stage and non-commercial research,
9. Better incentives to promote investment for new drugs and improving existing ones.

## The Critical Decision Continuum

The O’Neill Review recognizes that no single measure will solve the problem of AMR and only seeks to lay out a broad agenda. The review’s introduction emphasizes the inability of current diagnostic procedures to provide rapid and comprehensive answers, noting that it is

…incredible that doctors must still prescribe antibiotics based only on their immediate assessment of a patient’s symptoms, just like they used to when antibiotics first entered common use in the 1950s.

**Figure 1:**
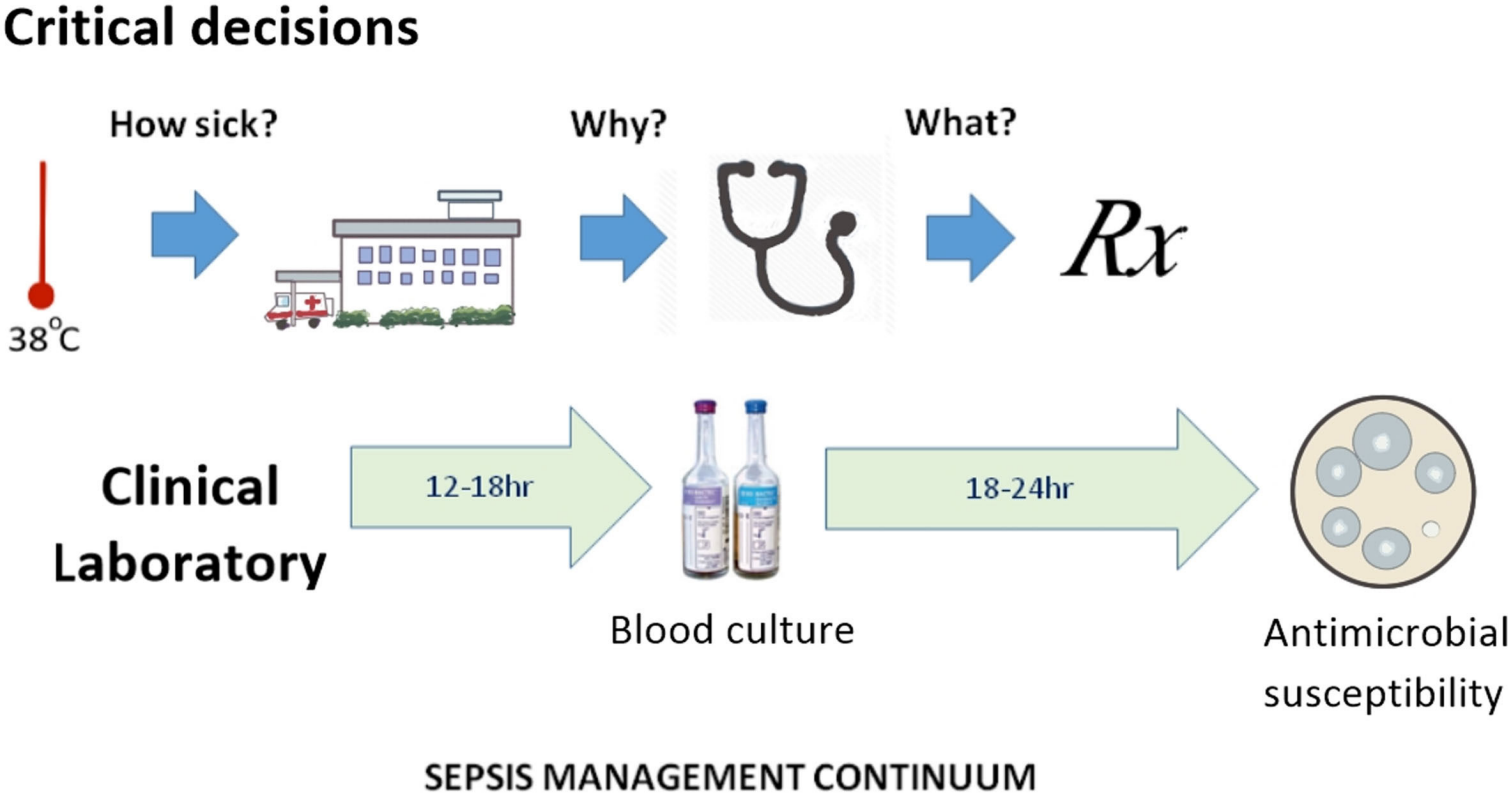
**The sepsis management continuum, showing alignment of time-critical clinical decision points with clinical microbiology laboratory data generation**.

Antibiotic prescribers face three major obstacles: (a) AMR is an abstract concept for all but its victims and their physicians; (b) detection of specific forms of AMR does not conclusively determine the best choice of anti-infective therapy; and (c) in severe infections, the wait for laboratory evidence on which to base a choice of antibiotic can have fatal consequences. This last consideration remains a key promoter of emerging AMR and could be described as poorly targeted personal medicine; the antithesis of precision public health. Half a millennium ago, Machiavelli observed that the increase in diagnostic certainty with the passage of time leads to reduced treatment success (19). This makes the physician reluctant to wait for the definitive culture results and subsequent antimicrobial susceptibility before commencing treatment. The clinical laboratory still relies on culture-based methods (20), despite continued interest in sepsis biomarker and other culture-independent technologies. The definition of sepsis has been a point of debate, since it rests on a range of non-specific clinical features and laboratory indicators. The most recent consensus statement on sepsis recognizes only two clinical categories (sepsis and septic shock) and recommends preliminary patient assessment with an easily applied clinical scoring method (qSOFA) (21). The three critical decision steps in the early stages of clinical management of sepsis occur before-, at-, and immediately after hospital admission, which approximate to determination of illness severity, its etiology and the choice of definitive therapy (Figure 1). From a precision public health perspective, these correspond to pre-hospital point of care tests that distinguish viral from bacterial infection, rapid hospital biomarker tests for sepsis, or culture-independent tests for severe viral infection and bacteremia and rapid determination of antimicrobial susceptibility. The greatest benefit is most likely to be a pre-hospital, rule-out test that distinguishes possible bacterial from viral infection (22). Improved speed and accuracy of bacterial detection and antimicrobial susceptibility testing has thus become a priority in managing the subsequent stages of sepsis and demands a culture-independent approach (23).

## Antimicrobial Susceptibility Tests

The mechanisms of AMR are numerous, increasing in variety, prevalence, and geographic distribution (24), but the ecological inevitability of AMR should not have caught us by surprise. Many antimicrobial agents are derivatives of naturally occurring compounds, whose corresponding AMR has its origins in the environment in which the antimicrobial compound evolved (25). However, the global success of a small number of multiresistant species such as *Klebsiella pneumoniae* (26) happened faster than predicted. The invisible, abstract nature of this public health threat is one of the more difficult aspects of the challenge we now face. It is unfortunate that the clinical laboratory markers of AMR do not translate into specific infectious diseases like septicemia, pneumonia, or meningitis. The bacterial species names that appear on public health notification lists are not by themselves notifiable diseases. Despite its limitations, the international standard method of antimicrobial susceptibility testing; broth microdilution minimum inhibitory concentration (MIC), converts the susceptibility of a particular bacterial isolate into a comprehensible measurement (27). The widely performed disc diffusion susceptibility test converts antimicrobial susceptibility into a visible and qualitative approximation to clinical outcome; sensitive or resistant. Disc diffusion and MIC tests, therefore, generate measurable and clinically valuable indicators of the antimicrobial effect against named bacteria, whereas resistance mechanism detection by nucleic acid amplification, gene sequencing, or other molecular means is not a reliable quantitative measure of antimicrobial sensitivity. The guidance these susceptibility tests give the prescriber in their choice of antimicrobial agent relies on a second growth step, which adds a further delay to the clinical laboratory process. Many prescribers are not interested in the specific identity of AMR mechanisms, particularly if the overall AMR phenotype is a combination of multiple molecular mechanisms, with varied *in vivo* expression and an unpredictable impact on clinical outcome. A carbapenem-resistant *K. pneumoniae* septicemia cannot be treated with a carbapenem, whether the mechanism of resistance is NDM-1, OXA-48, VIM, or IMP. The antimicrobial susceptibility phenotype is, therefore, a critical decider in the sepsis management continuum, even if the laboratory result comes 24–48 h after the initial choice of presumptive antimicrobial therapy. The susceptibility phenotype currently determines definitive therapy and ultimately informs the wider public health community. At present, surveillance data on antimicrobial susceptibility vary with laboratory capability, capacity, and locally determined public health priorities. These are all under-resourced, particularly in remote regional settings and in low-income countries (28). Nevertheless, multinational networks such as EARSS and CAESAR collect regional AMR data and interest is growing in standardizing the susceptibility tests on which surveillance relies (29–31). The monitoring task is easier when centers that combine a longstanding interest in sepsis and AMR collect prospective data from invasive infections (32).

## Emerging Laboratory Approaches to AMR

Rapid, culture-independent phenotypic tests are needed that improve precision in antimicrobial prescribing (17, 18). In particular, tests are needed that measure antimicrobial susceptibility, indicate effective treatment choices and deliver their results closer to the point of care. The wide diversity of molecular mechanisms of AMR limits the value of nucleic acid amplification (PCR assays) as a guide to antibiotic selection in acute clinical settings, particularly for carbapenem-resistant Gram-negative bacteria, which require supplementary tests to improve test sensitivity and overall coverage (33). Much effort has been devoted to detection of AMR mechanisms by rapid whole bacterial genome sequencing (3). Though this approach is not yet feasible as a routine service in the clinical laboratory, bacterial genome sequencing has clear application to public health investigations of AMR infection (3, 11, 26, 34), where decision triggers and task selection procedures can be applied to avoid overloading reference laboratory capacity. Clinical microbiologists who have to cope with the practical scientific challenge of detecting AMR while patients are still under treatment concentrate their effort on standardizing accurate measurement of the AMR phenotype (29). Faster methods of antimicrobial susceptibility testing are now a high priority, as noted in one of the O’Neill Review’s technical reports (35). It is here that systems biology applications are beginning to bear fruit (36). However, careful validation is necessary before emerging technologies can be used in the clinical laboratory. This requires test verification and harmonization to maximize analytical value and avoid poorly coordinated proliferation (29, 30). Systematic validation of new antimicrobial susceptibility test methods against agreed reference standards is a necessary step to delivering sufficient confidence in emerging laboratory methods before they can be used for surveillance and control purposes. High profile incentives such as the UK Longitude Prize are being used to attract new candidate tests for this lengthy development process (37).

## A Blend of Countermeasures

**Figure 2:**
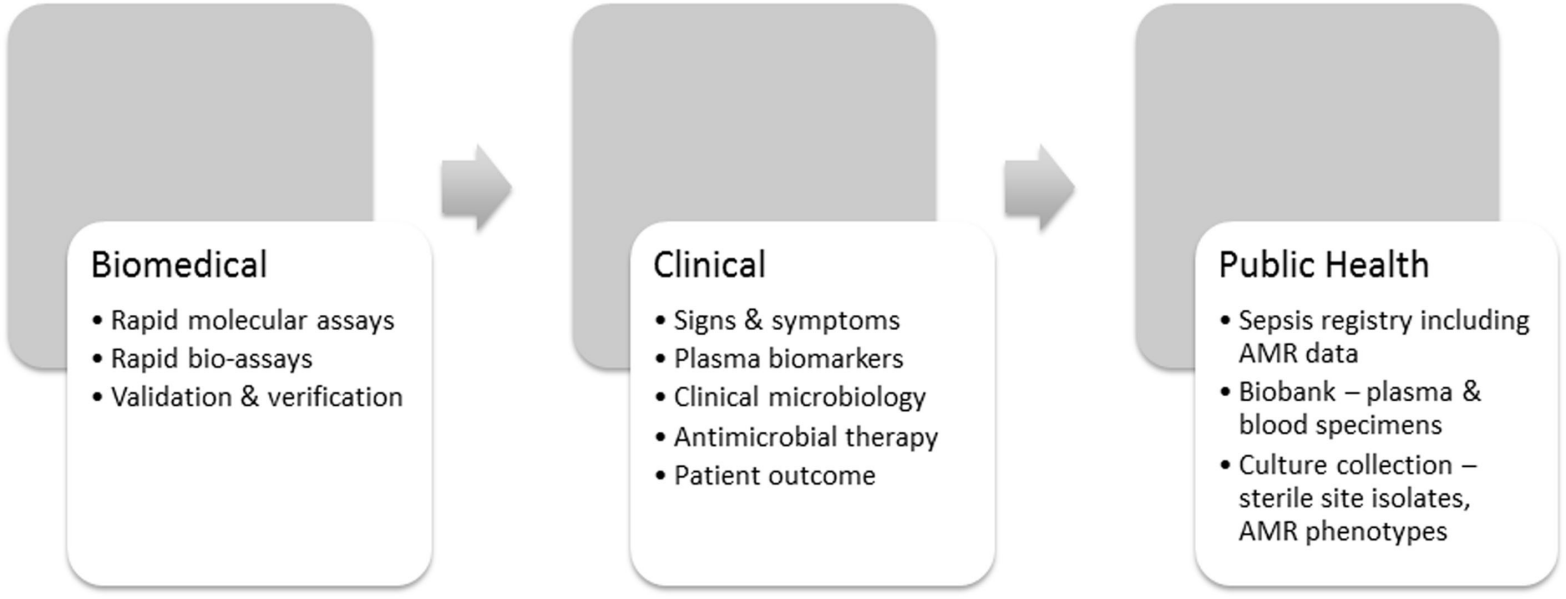
**Technical progression in support of AMR/sepsis countermeasures**.

Countermeasures need purpose, intent, direction, and evidence for their efficacy. An understanding of the complex intersection of laboratory, clinical, and public health insights will improve their beneficial effect (16). AMR-specific countermeasures, therefore, operate at three levels (Figure 2) beginning with faster and more accurate phenotypic laboratory assays that use agreed international standards (29, 30, 36). The O’Neill Review expects new laboratory technology to enable recognition of sepsis, its etiology and antimicrobial susceptibility faster than current culture-dependent methods (35). At the clinical level, prescribing physicians need incentives such as faster confirmation of the etiology of infection and its antimicrobial susceptibility to use the evidence-based antimicrobial therapy advocated in the O’Neill Review (18). In addition to the recommended clinical sepsis score (21), prescribing physicians need a bacterial infection rule-out test to support their initial sepsis triage (22) and innovative methods of rapid antimicrobial susceptibility testing to support their decision-making at the point of care. However, a clearer picture of the global burden of AMR and the measures to control it will not emerge until variations in regional AMR notification have been harmonized through introduction of a sepsis/AMR registry (Figure 2). Other fields of medicine, such as oncology, use case registries to develop and refine their disease-specific countermeasures (38, 39). A sepsis registry could be used in similar manner as a precision public health tool to stratify sepsis by syndrome, etiology, AMR phenotype, and resistance mechanism, and, therefore, to coordinate AMR countermeasures. The recent consensus definition of sepsis is a helpful starting point for discussion of a sepsis registry (21), but requires a stronger laboratory-based emphasis on bacterial etiology and AMR. Precision is measurable, particularly when supported by archival material in bacterial culture collections and registered clinical biobanks. Claims for the increased accuracy of new methods should thus be verifiable and linked with the clinical laboratory, where the precision of antimicrobial susceptibility tests is already monitored against reference standards and verified by regulatory agencies (29, 30).

## Conclusion

Antimicrobial resistance has become a global tragedy of the commons, driven by a complex bacterial survival trade-off at a cellular level (40). Now that AMR is recognized as a global priority, it is time to learn to use additional systems biology tools to improve the speed and accuracy of antimicrobial prescribing at an individual patient level and simultaneously increase the precision of AMR sepsis surveillance. Improved confidence in the recognition of early sepsis, faster determination of its etiology, and antimicrobial susceptibility phenotype, and real time surveillance through an AMR sepsis registry will lead to more effective coordination of clinical, laboratory and public health AMR countermeasures. Given the speed with which antimicrobial agents have been compromised by AMR, there is no time to lose introducing these laboratory and surveillance tools into wider use.

## Author Contributions

The authors are working together on culture-independent pathology test development. TI prepared the initial draft. NU reviewed, edited, and supplemented the first draft with an emphasis on sepsis. Subsequent versions of the manuscript were exchanged between the authors who both approved the final version.

## Conflict of Interest Statement

The authors are supported by a Grand Challenges award from the Bill and Melinda Gates Foundation, as stated in the acknowledgments above. This and research translation grants from the Government of Western Australia are being used to develop culture-independent pathology tests for sepsis and AMR countermeasures. Thermo Fisher Scientific and Biomerieux have provided in-kind support to the authors’ research group, under supervision of the WA Health Department’s Research Development Unit. Neither author has received funding from these companies for any purpose. No supporting organization or its members had any role in the preparation of this manuscript, which is the opinion of the two authors.
